# Riboswitches as hormone receptors: hypothetical cytokinin-binding riboswitches in *Arabidopsis thaliana*

**DOI:** 10.1186/1745-6150-5-60

**Published:** 2010-10-20

**Authors:** Jeremy Grojean, Brian Downes

**Affiliations:** 1 Saint Louis University, Department of Biology, 235 Macelwane Hall, 3507 Laclede Ave St. Louis, MO 63103, Saint Louis, MO 63110, USA; 2 Saint Louis University, Department of Biology, 226 Macelwane Hall, 3507 Laclede Ave., St. Louis, MO 63103, USA

## Abstract

**Background:**

Riboswitches are mRNA elements that change conformation when bound to small molecules. They are known to be key regulators of biosynthetic pathways in both prokaryotes and eukaryotes.

**Presentation of the Hypothesis:**

The hypothesis presented here is that riboswitches function as receptors in hormone perception. We propose that riboswitches initiate or integrate signaling cascades upon binding to classic signaling molecules. The molecular interactions for ligand binding and gene expression control would be the same as for biosynthetic pathways, but the context and the cadre of ligands to consider is dramatically different. The hypothesis arose from the observation that a compound used to identify adenine binding RNA sequences is chemically similar to the classic plant hormone, or growth regulator, cytokinin. A general tenet of the hypothesis is that riboswitch-binding metabolites can be used to make predictions about chemically related signaling molecules. In fact, all cell permeable signaling compounds can be considered as potential riboswitch ligands. The hypothesis is plausible, as demonstrated by a cursory review of the transcriptome and genome of the model plant *Arabidopsis thaliana *for transcripts that *i) *contain an adenine aptamer motif, and *ii) *are also predicted to be cytokinin-regulated. Here, one gene, *CRK10 *(for *Cysteine-rich Receptor-like Kinase 10*, At4g23180), contains an adenine aptamer-related sequence and is down-regulated by cytokinin approximately three-fold in public gene expression data. To illustrate the hypothesis, implications of cytokinin-binding to the *CRK10 *mRNA are discussed.

**Testing the hypothesis:**

At the broadest level, screening various cell permeable signaling molecules against random RNA libraries and comparing hits to sequence and gene expression data bases could determine how broadly the hypothesis applies. Specific cases, such as *CRK10 *presented here, will require experimental validation of direct ligand binding, altered RNA conformation, and effect on gene expression. Each case will be different depending on the signaling pathway and the physiology involved.

**Implications of the hypothesis:**

This would be a very direct signal perception mechanism for regulating gene expression; rivaling animal steroid hormone receptors, which are frequently ligand dependent transcription initiation factors. Riboswitch-regulated responses could occur by modulating target RNA stability, translatability, and alternative splicing - all known expression platforms used in riboswitches. The specific illustration presented, *CRK10*, implies a new mechanism for the perception of cytokinin, a classic plant hormone. Experimental support for the hypothesis would add breadth to the growing list of important functions attributed to riboswitches.

**Reviewers:**

This article was reviewed by Anthony Poole, Rob Knight, Mikhail Gelfand.

## Background

### Aptamers

Aptamers are nucleic acids capable of binding a specific ligand. They are frequently isolated using a SELEX (for Systematic Evolution of Ligands by EXponential amplification) screen. Briefly, in a SELEX Screen random nucleic acid oligos are incubated with an immobilized ligand and washed to allow removal of non-binding sequences. The bound fraction is then expanded by *in vitro *transcription and reselected on the ligand column. This process is repeated until only those nucleic acid species with high affinity for the ligand remain [1]. Through this reiterative process aptamers that bind proteins, small molecules, and even other nucleic acids sequences have been identified [2]. This process has led to the generation of aptamer molecules with a wide range of research and diagnostic capabilities [3,4].

### Riboswitches

Riboswitches are elements found in mRNA that are capable of altering gene expression upon the direct binding of a ligand. Ligand binding to a riboswitch is not dependant on protein intermediates. Riboswitches have been implicated in the metabolic regulation of diverse biosynthetic pathways ranging from the purines adenine [5] and guanine [6], the cofactors riboflavin [7], thiamine [8], and cobalamin [9], the amino acids lysine [10] and methionine [11], and glucosamine-6-phosphate [12] among others. Prokaryotic riboswitches are capable of altering gene expression by causing early termination of transcription or preventing translation [7]. A novel role in regulation has been described wherein the riboswitch has ribozyme activity that cleaves the mRNA in which it resides [12]. Furthermore, although riboswitches have not yet been found to directly regulate signal transduction, this may soon change. Characterization of the *ydaO *structured RNA element, found commonly in gram-positive bacteria, suggests that prokaryotes may indeed use riboswitches to regulate their physiological responses [13]. In addition, the temperature-dependent stability of the *E. coli *cold shock *cspA *transcript is a fascinating example of a structured RNA element sensing the environment to regulate gene expression [14].

Riboswitches are composed of two functional domains, a metabolite binding region, or aptamer domain, and an expression platform. Portions of the expression platform can base pair with portions of the metabolite binding region depending on the presence or absence of metabolite. It is these changes in base pairing upon metabolite binding that lead to structural changes in the mRNA, which in turn lead to the regulatory effects [15]. The riboswitches of bacteria and archeabacteria are regulators of synthesis pathways [5-12], perhaps operative in 2% or more of all bacterial genes [6].

The recent identification of riboswitches in eukaryotes has expanded the concept of the expression platform. In almost all cases bacterial riboswitch elements are located in the 5' untranslated region of mRNAs [15] including TPP (thiamine pyrophosphate) binding riboswitches. TPP binding riboswitches have been identified in *Arabidopsis thaliana *and *Neurospora Crassa *[16,17]. In these two examples, the location of the riboswitch is different from prokaryotes suggesting divergence of the expression platform despite conservation of the TPP binding sequence. In Arabidopsis the riboswitch is located in the 3' untranslated region of the *THIC *gene, just 5' of the polyA tail [18]. The Neurospora TPP riboswitches are located within the first and second introns of the *NMT1 *and *THI4 *genes, respectively [17]. In both Neurospora and Arabidopsis, ligand binding causes a conformational change and alternative splicing of the transcripts [17]. In Neurospora, TPP-binding leads to intronic translation and a premature termination codon, whereas in Arabidopsis, TPP-binding causes splicing of the consensus polyadenylation sequence [17,18]. This leads to the production of several polyadenylation variants which have less stability than transcripts polyadenylated at the conserved site [18]. Despite these radical departures from the model prokaryotic riboswitch expression platform, it remains that this singular example of a eukaryotic riboswitch is involved in the regulation of a biosynthetic pathway [16]. The mechanistic divergence between prokaryotic and eukaryotic riboswitches leaves open the possibility that functional divergence has occurred as well. As life became more complex riboswitches could have evolved to play a broader role in signal perception, transduction, and integration.

### The Cytokinins: N^6^-substituted Adenine Analogs

Cytokinins are plant hormones, also called plant growth regulators. Cytokinins play a diverse and highly integrated role in plant growth and development, but are traditionally considered compounds that promote cell division and growth [19]. Roles for cytokinins include bud formation, leaf growth, shoot organogenesis, cotyledon expansion, chloroplast development, nutrient mobilization and leaf senescence [20,21], among others.

**Figure 1:**
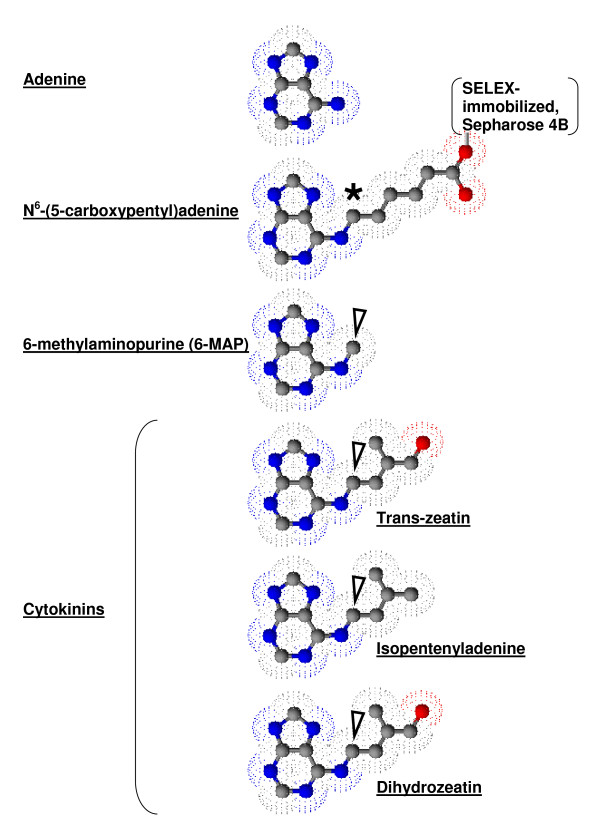
**Structure of Adenine and related compounds, N^6^-(5-carboxypentyl)adenine, 6-methylaminopurine, and naturally occurring cytokinins**. Adenine (top) is compared to the SELEX matrix N^6^-(5-carboxypentyl)adenine-Sepharose 4B (below). Electron density maps (speckles) indicate the surface RNA could interact with around the ball and stick structures of carbon (grey), nitrogen (blue) and oxygen (red). The black asterisk indicates the N^6 ^substitution anchoring adenine to the SELEX matrix. N^6^-substituted carbon is also found in the remaining structures (arrows) including 6-methylaminopurine (6-MAP), shown by Meli et al. (2002) to bind N^6^-(5-carboxypentyl)adenine-Sepharose 4B selected aptamers with higher affinity than adenine. Three major naturally occurring cytokinins are also included.

The most prevalent naturally occurring cytokinin, zeatin, and a number of other isoprenoid class cytokinins including isopentyladenine, and dihydrozeatin are N^6^-substituted adenine analogs (Figure 1). Chemically, these cytokinins are adenine with an N^6 ^substituted hydrophobic isoprene adduct. Cytokinins are found as modified bases in tRNA, as a nucleoside bound to ribose, as mono-, di-, and tri-phosphorylated nucleotides, and as a free base. The free base is the biologically active form [22]. Their production has been identified in soil amoeba [23] and the bacteria *Agrobacterium tumefaciens *[24]. It was suspected that bacteria were responsible for the production of plant cytokinins until the biosynthetic enzyme adenylate isopentenyltransferase (IPT) was characterized in plants [20,25]. Cytokinins are largely produced in the roots and transferred by the xylem to apical tissues where they exert their effects [26].

A genetic screen for cytokinin induced shoot production in tissue culture identified *CRE1/AHK4 *(*cytokinin response *1/Arabidopsis His Kinase 4) as a cytokinin receptor [27]. It is a histidine kinase which participates in a His-Asp phosphorelay similar to the bacterial two-component system. The homologous histidine kinases AHK2 and AHK3 also show cytokinin dependent histidine kinase activity [28]. Ultimately, all three receptors lead to the activation of the ARR (Arabidopsis Response Regulators) protein family to regulate gene expression. This histidine phosphorelay system does not preclude signaling of cytokinins through other mechanisms/receptors. It is reasonable that parallel signaling systems exist to modulate and fine-tune the many responses attributed to cytokinins.

We propose that riboswitches function as receptors for signaling molecules, including the classic plant hormone cytokinin. As with many known receptor signaling pathways, the end result would be altered gene expression, but with a riboswitch the effect would be direct and instantaneous. We suggest that by comparing riboswitch binding metabolites to chemically related signaling molecules riboswitches dedicated to signal transduction might be revealed. Here, we consider the SELEX obtained sequence for adenine binding aptamers, obtained using the N^6 ^substituted adenine analog N^6^-(5-carboxypentyl)adenine, a close structural relative to the plant hormone cytokinin (Fig.1). Through a review of public Arabidopsis genome resources, we conclude that it is indeed plausible that cytokinin-binding riboswitches exists in the Arabidopsis genome, and that such switches could serve as a currently undescribed class of plant hormone riboswitch receptors.

## Presentation of the Hyposthesis

The hypothesis presented here is that hormones are perceived by riboswitches to integrate or initiate signal transduction cascades in eukaryotes. To explore the idea, known metabolite-regulatory aptamers and riboswitches were surveyed for related molecules with known roles in cell signaling. In particular, adenine and the class of plant growth regulators called cytokinins were selected to illustrate the hypothesis.

Meli et al. (2002) isolated adenine binding aptamers by cleverly designing a SELEX experiment to avoid modification of the adenine imidazole ring that is recognized in a variety of biological reactions. The adenine N^6 ^position was substituted with a 5-carboxypentyl group that could be tethered to sepharose for SELEX screening (Figure 1). After identifying several aptamer sequences capable of binding adenine, the highest affinity aptamers were assayed with molecules structurally similar to adenine. Interestingly, 6-MAP (6-methylaminopurine) bound with higher affinity than adenine itself [29], perhaps reflecting the N^6^-linked adenine configuration used in the SELEX screen. 6-MAP has a methyl group at position N^6 ^similar to both the SELEX matrix used for aptamer selection and to naturally occurring cytokinins (Figure 1). Thus, although prokaryotic adenine-binding riboswitches have now been extensively characterized [30-32] we focused on these artificial N^6^-(5-carboxypentyl)adenine binding aptamers. None-the-less, it would be interesting to examine plant transcriptomes for prokaryote-related adenine riboswitches, especially as bioinformatics tools for such analysis are rapidly being developed [33-35].

### Potential Cytokinin-Binding Riboswitches

To evaluate the plausibility of the hypothesis, three existing data sets were compared including *i) *the ten nucleotide core from the N^6^-(5-carboxypentyl)adenine binding aptamers described by Meli et al. (2002), *ii) *the Arabidopsis genome, and *iii) *microarray data of cytokinin-treatment experiments. Here we make the assumption that a cytokinin-binding riboswitch would change gene expression in a cytokinin-dependent fashion. Clearly there are possible expression platforms that would not alter transcript level, but adding this criterion focused our attention and hypothesis on a narrow group of genes indeed known to be cytokinin-regulated.

Nucleotide BLAST searches[36] were performed using four aptamers identified by SELEX screens to have high affinity for N^6^-(5-carboxypentyl)adenine [29]. Arabidopsis sequences with a match to seven out of ten nucleotides in the core were classified as either coding or non-coding sequence and retained as candidate aptamers (CaAps) for further consideration. Since the spacing of the nucleotides was also a criterion, only 63 genes were retained.

Independently, microarray data was obtained from the Nottingham Arabidopsis Stock Centre [37]. The data for wild-type *Col-0 *seedlings 3 hour cytokinin-treated and untreated [37, Expt. No. 181] revealed 2,867 genes regulated with a two-fold expression change upon cytokinin treatment.

**Figure 2:**
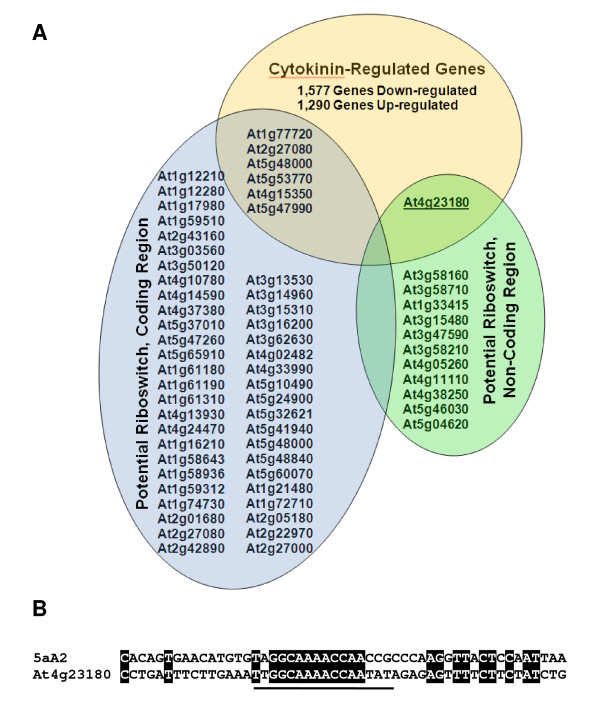
**Candidate cytokinin-binding riboswitch aptamers (CaAps)**. Three data sets; N^6^-(5-carboxypentyl)adenine selected aptamers, the Arabidopsis genome/transcriptome, and cytokinin microarray expression data are presented in A) as a Venn Diagram. Genes up- or down-regulated by cytokinin at least two-fold are summarized in light orange. Genes with a match to seven out of ten nucleotides in the N^6^-(5-carboxypentyl)adenine selected aptamer conserved core domain (underlined in B) are in light blue or green. Due to the short query sequences (each aptamer was 50 nt), and having few bases conserved outside the core, it was not possible to narrow search results using statistical cut-offs. Rather, low stringency search parameters were used (Expect Threshold: 1000, Word Size: 7, Match/Mismatch: 1,-3) to identify CaAps (Expect Threshold: 1000, Word Size: 7, Match/Mismatch: 1,-3). The first reason for this was to retrieve all possible binding sequences for further analyses. The second reason is because while the ligand-binding core is probably conserved, other nucleotides in the riboswitch need only form proper base-pairing structures and could have higher sequence heterogeneity. Both the non-redundant nt collection and RefSeq mRNA databases were searched limiting results to *Arabidopsis thaliana *(taxid: 3702). Approximately 100 nucleotides on either side of the CaAp sequences were retrieved from the NCBI (National Center of Biotechnology Information) core nucleotide entry. NCBI and TAIR (The *Arabidopsis *Information Resource) databases were used to map sequences to a location in the *Arabidopsis *transcriptome and classified as Coding (light blue) or non-coding (light green). One gene At4g23180, *CRK10 *(underlined in A) was selected as an illustration of the hypothesis because it is down-regulated three-fold by cytokinin, it is in an intron, and, as depicted in B) its sequence is conserved with the 5aA2 aptamer [28] throughout, and especially in the core domain (underlined).

**Figure 3:**
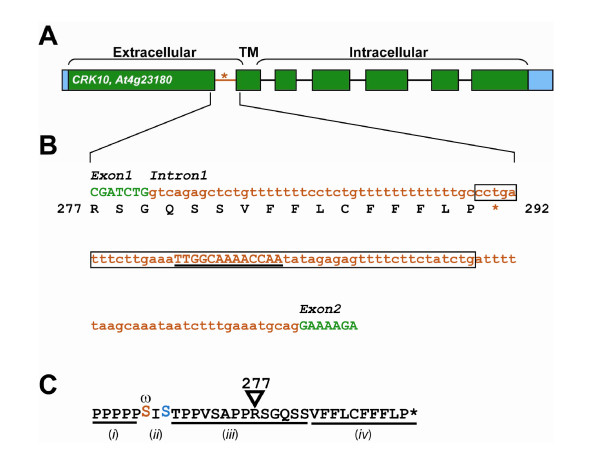
**Structure of CRK10**. A) Boxes represent untranslated and exonic regions, blue and green, respectively. A connecting line represents introns where the red section is the CaAp-containing intron 1. A red asterisk indicates the PTC introduced by intron 1 retention. Exon 2 encodes the transmembrane (TM) α-helix that separates Extra- from Intra-Cellular domains of the protein (as indicated). B) Intron 1 detail is shown. Uppercase green letters are exon sequence, as indicated, and red lowercase letters are intronic with predicted amino acid sequence shown below and culminating in the PTC (red asterisk). The CaAp region related to the 5aA2 aptamer is boxed. The core of the potential riboswitch sequence is underlined. C) The alternative transcript of *CRK10 *has a consensus for modification with a GPI-anchor at the ω position. The consensus includes (*i*) a string of prolines N-terminal to the ω residue is which are likely hydroxylated; (*ii*) required residues at the ω and ω+2 positions (red and blue respectively); (*iii*) a flexible moderately polar but uncharged region; and (*iv*) the final C-terminal string of non-polar residues heavy in aromatic amino acids [52,53].

Combining the sequence and expression data sets, seven sequences were found to meet all of the above criteria (Figure 2A). Six of these sequences were in coding regions, and one, the *Cysteine-rich Receptor-like Kinase *gene (*CRK10*, At4g23180), was non-coding (Figure 3AB). The *CRK10 *transcript is 3-fold down-regulated by cytokinin and contains a CaAp in the first intron of the pre mRNA with 5A2 sequence conservation in and around the aptamer core (Figure 2B). Eukaryotic riboswitches characterized to date have been found in non-coding regions and use alternative splicing based expression platforms, therefore the *CRK10 *gene was selected as an example to discuss how a cytokinin receptor/riboswitch could hypothetically function to influence signal transduction pathways.

### A Hypothetical Cytokinin-Binding Riboswitch Containing Gene

*CRK10 *encodes a Receptor-Like Kinase, or RLK; an important class of plant receptors. RLKs are analogous to Receptor Tyrosine Kinases in animals; except that they usually auto- (trans-) phosphorylate serine or threonine residues instead of tyrosines. RLKs, including CRK10, have a putative extracellular ligand binding domain, a single pass transmembrane α-helix, and an intracellular kinase domain that is activated upon ligand-dependent receptor dimerization. Within the large Arabidopsis RLK family, there are 42 predicted *CRKs *[38]. *CRK10*, originally designated *RLK4*, was first identified as a pathogen-response gene transcriptionally activated by WRKY transcription factors [39]. However, ligands for, and the signal transduction cascade initiated by CRK10 are currently unknown. It is also not known whether CRK10 is activated by homodimerization, or if it can heterodimerize with the 41 other CRKs in a combinatorial receptor system. The ability of pathogens to co-opt and utilize plant cytokinin signaling [40-42] raises interesting possibilities regarding a cytokinin binding riboswitch in a pathogen response pathway.

For the sake of discussion, we assume that the CaAp in CRK10 does indeed contribute to a cytokinin-riboswitch, in which case it would be likely to regulate alternative splicing based on known examples. For instance, the thiamine-pyrophosphate (TPP) riboswitch identified in *Arabidopsis thaliana*, the only plant riboswitch discovered to date, masks a 5' splice site in the 3' UTR of the *THIC *gene leading to alternative polyadenylation[18]. The potential riboswitch in *CRK10 *is in the middle of the first intron in a position where changes in RNA structure could block the binding of the snRNP U2. This would prevent proper formation of the spliceosome. Retaining the first intron in the transcript would cause two things to happen, *i) *the first 278 residues would be translated from exon 1, fused to an alternate 14 residues encoded by retained intron 1, and *ii) *a Premature Termination Codon (PTC) would prevent further translation. In essence, only the extracellular portion of the protein would be expressed, stopping 6 residues N-terminal to the transmembrane α-helix (Figure 3AB).

Two alternative implications of *CRK10 *intron1 retention hinge on whether alternative cleavage and polyadenylation occurs. Without cleavage and polyadenylation, the transcript would contain five Exon Junction Complexes (EJCs) downstream of the stop codon and possibly signal Nonsense Mediated Decay (NMD) of the transcript. If cleavage and polyadenylation occurs in intron or exon 1 then additional 3' EJCs and NMD would be eliminated allowing expression of the truncated protein. The microarray data did not allow us to discriminate between these possibilities because in either case the 3' *CRK10 *sequence hybridizing to the Affymetrix array would be down-regulated. Ultimately, the functional consequence of either model offers an intriguing discussion scenario for a hypothetical cytokinin-riboswitch expression platform.

NMD, in the absence of cleavage and polyadenylation could be used in a regulatory mechanism called RUST (for Regulated Unproductive Splicing and Translation). In RUST, alternative splicing produces a stop codon that initiates NMD of the transcript [43]. RUST has been proposed as an autoregulatory mechanism for proteins involved in splicing reactions such as *C. elegans *SRp20 and SRp30b mRNAs [44,45], and for transcripts that have an extremely long synthesis time such as dystrophin, which cannot realistically be regulated at the transcriptional level [43].

However, cleavage and polyadenylation of an intron retained version of *CRK10 *is plausible. Intron 1-retained (IR) *CRK10 *transcript contains a putative cleavage and polyadenylation signal in the 3' end on the retained intron. Specifically, a near upstream element AAUAAU sequence is located 11 nucleotides upstream of a potential cleavage site CA (Figure 3B). Approximately 50 nt upstream of the potential cleavage site is a potential far upstream element that is U-rich. These sequences are consistent with loosely conserved plant polyadenylation signals[46]. In the relatively well characterized human transcriptome approximately 20% of all human transcripts present alternative intronic polyadenylation events[47] suggesting that coordinated splicing and polyadenylation rescues certain eukaryotic transcripts from NMD. This appears to be conserved in the plant kingdom as well, 50% of studied rice transcripts have more than one polyadenylation site, and 4% of rice genes have alternative polyadenylation in 5' UTRs, introns, or protein coding regions[48].

Stable *IR CRK10 *would encode the original extracellular domain appended to a novel C-terminus. Examination of this predicted C-terminus identified a strongly predicted plant glycosylphosphatidylinositol (GPI) anchor sequence in lieu of the transmembrane sequence found in the full length protein (Figure 3C). GPI-anchoring is known to localize proteins to lipid rafts in the plasma membrane. The extracellular domain of CRK10 contains two DUF26 domains (domain of unknown function). These domains have been found both in transmembrane receptors and secretory proteins. It is known that two proteins containing this domain are GPI-anchored[49].

**Figure 4:**
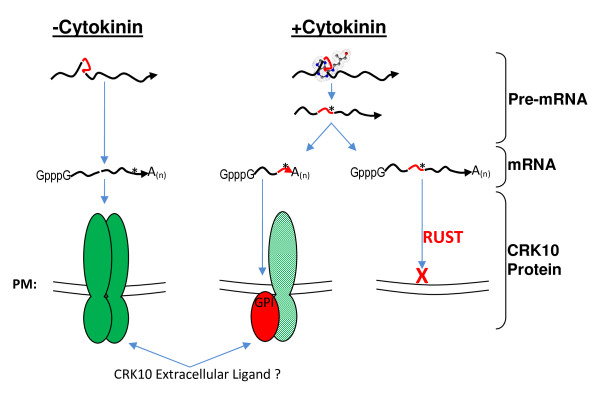
**Hypothetical Models of CRK10 Regulation by Cytokinin**. Pre-mRNA and mRNA are depicted by wavy black arrows with a red segment indicating intron 1, or a gap indicating removal of intron 1. Blue arrows indicate the flow of information. Positions of capping or cleavage and polyadenylation are shown as GpppG and A_(n)_, respectively. Parallel lines represent the plasma membrane (PM) with the cell exterior at the bottom.

Taken together, a speculative model of *CRK10 *riboswitch-dependent processing is used to illustrate our hypothesis. Highlighted are mechanisms integrating cytokinin perception with regulation of CRK10 signaling (Figure 4). First, the cytokinin-binding riboswitch would, at a minimum, prevent expression of the protein kinase domain by promoting retention of a stop codon containing intron. mRNA surveillance mechanisms could then destroy the transcript in the NMD pathway. If however, alternative splicing were accompanied by alternative polyadenylation, a transcript encoding a GPI-anchored extracellular domain only protein could serve as a receptor decoy on the outer leaflet of the plasma membrane. This decoy receptor could dimerize with full-length, functional CRK10 in lipid rafts preventing activation of kinase activity. The decoy receptor could also compete with the full-length receptor for ligand binding. Thus, this speculative model presents a plausible example of a riboswitch controlling expression of the CRK10 protein at all levels from transcription to protein activity. At its core, this model depicts the immediate molecular changes to signal transduction that could occur upon perception of cytokinin by a riboswitch.

## Testing the Hypothesis

Our general hypothesis that hormones can be perceived by riboswitches to directly control gene expression and signal transduction could be approached in at least two ways. First, published SELEX screens may reveal chemically related motifs, much like the similarity between adenine, N^6^-(5-carboxypentyl)adenine, and cytokinin used to frame the current hypothesis. In our own brief survey, we did not find other significant matches beyond cytokinin, but a more comprehensive search, and future SELEX screen results should be considered. Second, additional SELEX screens designed to directly isolate hormone binding aptamers could be conducted. Sequences discovered in these screens could reveal endogenous regulatory systems when compared to the growing list of complete genomes, or alternatively, be used to engineer sensitive hormone sensors. Such sensors could be versatile tools in model organisms because they would be expressed from transgenic DNA.

Our specific illustrative hypothesis regarding *CRK10 *could be tested in several defined ways that would generally apply to the characterization of hormone perception by riboswitches. The first step in testing the hypothesis would be to determine whether *CRK10 *mRNA transcripts bind cytokinin *in vitro *using an in-line probing approach. In this assay structural changes in the RNA, induced by cytokinin binding, could be detected in the degradation pattern of end-labeled *CRK10 *RNA. To establish biological specificity and relevance, the affinity of the riboswitch for *bona fide *cytokinins would be compared to inactive analogs. These assays are now well established for the characterization of riboswitches [7,50,51].

Established *in vitro *binding would be followed with RT-PCR and 3' RACE assays to probe cytokinin-induced changes to endogenous *CRK10 *RNA structure. Analysis would initially focus on alternative-splicing and retention of the first intron, and on alternative polyadenylation events.

qRT-PCR would be used to confirm the microarray data that CRK10 mRNA abundance drops three-fold upon cytokinin treatment. Primer sets specific to a truncated and a full length *CRK10 *mRNA would be used to determine whether cytokinin-induced down-regulation of the entire mRNA occurs, which would support the NMD and RUST model. Alternatively, downregulation of only the sequence 3' of intron 1 would support the alternative polyadenylation and translation model. Ultimately, CRK10 protein would need to be examined using antibodies specific to the amino terminal extracellular domain. Immunoblotting experiments could directly test the two alternative models proposed here including *i) *the synthesis of a truncated CRK10 protein, and *ii) *the abundance of full length CRK10 protein.

Ultimately, activity of the protein product of a hormone riboswitch-containing transcript is key to interpreting the effect of hormone binding on signal transduction. For instance, the complete picture of *CRK10 *gene regulation will be hard to fully appreciate until its function as a protein, presumably including extracellular ligand binding, signaling, and effectors, are characterized. A preliminary study implicated *CRK10 *in the pathogen response [39]. Could CRK10 signaling arrests the cell cycle to control the spread of infection? This would be in direct opposition to cytokinin's general role in promoting growth and cell division. It is plausible that riboswitches could provide one means of integrating such opposing pathways.

## Implications of the Hypothesis

This hypothesis implies the expansion of riboswitch function beyond metabolite sensing and modulated synthesis pathways to include hormone perception and signal transduction. All of the pieces are in place; it is only a question of context. Riboswitches are known to bind small molecules to alter gene expression. If the small molecule is a cell permeable signaling compound, then, through a riboswitch, it could have an immediate impact on gene transcription in progress.

Since riboswitches receptors would act initially at the nucleotide level, there are even more possibilities for regulation than exist for protein receptors. A riboswitch receptor could directly influence the rapidly expanding list of regulatory mechanisms involving non-coding RNAs. For example, by regulating the expression or processing of miRNAs, hormone-binding riboswitches could change gene expression profiles on a large scale.

At an intermediate level, riboswitches could regulate protein coding RNAs as in the example posed for *CRK10*. Through alternative splicing the level of expression and even the primary protein structure can be controlled. In these cases, the form and level of the protein product is the first step in the signal transduction cascade. In the event that the target mRNA encodes a signaling molecule, as for CRK10, signal integration could occur. In this example, both nuclear perception of cytokinin and extracellular perception of a currently unknown ligand both impact the final activity of the intracellular CRK10 kinase domain.

In reality the number of conceivable expression platforms is virtually limitless. It seems likely that nature has bent the versatility of riboswitches to a useful purpose in eukaryotic signal transduction. Thus, it is essential that further studies be carried out to experimentally determine the breadth of riboswitch ligands.

## Competing interests

The authors declare that they have no competing interests.

## Authors' contributions

BD laid the foundation for the generation of the hypothesis. JG performed the bioinformatic review. JG and BD developed the final, hypothetical model and wrote the manuscript.

## Author's Response

We are grateful for the thoughtful comments provided by our reviewers. We believe that the caution raised by these critiques, coupled with our illustration of the idea, frame the work well. A number of important bioinformatics studies including natural adenine aptamer based searches, RNA fold-modeling, consultation with the protein, alternative splicing, and gene ortholog databases, as suggested in review, would be essential for any group further considering this idea. Experimentation would of course be the surest approach as indicated in the text and in review. Here we wish to reiterate to any group considering this hypothesis that a complete bioinformatic analysis would be wise before embarking on experimentation. Meanwhile, all of our annotated files are available upon request.

The emphasis of riboswitch research on metabolic signaling is not surprising considering the original discovery of riboswitches in that field, and the wealth of clear examples described in that context. Here, we suggest that a diversity of small signaling compounds might interact with riboswitches. In review it was well put that the difference is arbitrary - but this is an important aspect of our hypothesis. We do not argue a fundamental difference between how metabolic or developmental signaling molecules would interact with a riboswitch, we argue context. Indeed there are an astounding number of candidate compounds. In our case, the fact that an RNA sequence (albeit an artificial one) could bind to a cytokinin-like ligand was a compelling observation that had not been noted in the existing literature. It will be interesting to see if the functional breadth of riboswitches expands in years to come. If not, it will be interesting to learn what is special about metabolites and their riboswitches that makes the relationship exclusive.

## Reviewer'S Comments

### Reviewer 1: Anthony M. Poole

This manuscript reports a meta-analysis aimed at trying to identify candidate cytokinin-riboswitches. The authors began with four published SELEX-derived sequences identified as capable of binding N6-(5-carboxypentyl)adenine because this molecule is similar to cytokinins. They then screened the Arabidopsis genome for sequences that match the core aptamer sequence at 7 of 10 nucleotides. Next they scanned expression data sets for sequences that both carry candidate aptamer sequences and which show a two-fold expression change following cytokinin treatment. They identify seven expressed sequences, one of which was present in the fist intron of the CRK10 gene. The authors then develop a hypothesis on the possible existence of cytokinin riboswitches.

The premise here is that the aptamer sequences that have elsewhere been shown to bind adenine-related molecules will be associated with cytokinin binding in plants. This requires that the primary sequence is a sufficiently strong signal that identification of such sequences in genes that show altered expression can be used to identify plausible cytokinin-riboswitch candidates. This hypothesis is certainly possible, and the results of the analyses are intriguing. However, this really is a case where the proof is in the pudding - there is every chance that the CRK10 aptamer is a false positive, and what is really required is experimental analysis.

The authors provide no indication as to whether any available expression data are consistent with their hypothesis. A quick examination of cDNA data for this gene on tair.org (and in a few other datasets) did not reveal any evidence for alternative transcripts consistent with the model described, and I found no 2D proteomics data supporting the existence of a truncated protein product, likewise predicted by the model. My screens were in no way complete and are therefore not evidence against this hypothesis, but given that the analyses presented can at best only be considered circumstantial evidence, it would seem more productive for the authors, one of whom is an experimental molecular biologist working with Arabidopsis, to pursue an experimental approach to this question rather than presenting a preliminary set of meta-analyses. Given that many of the experimental tests the authors propose are routine in molecular biology labs, initial tests seem well within their expertise.

In lieu of experimental data, I think an examination of the false-positive discovery rate is also essential. Possible ways to get a sense of false-positive rates might be to repeat the search using shuffled or random aptamer sequences (the reverse, i.e. genome shuffling, may be trickier as intron-exon structure and CDS integrity would need to be retained). Another would be to randomly re-label equivalent sized sets of expressed sequences as cytokinin-regulated, and rescreen.

Against this backdrop of uncertainty over the validity of the aptamer sequence screen, if the authors are able to find evidence of evolutionarily conserved candidate cytokinin-riboswitches, that would certainly add some weight to their proposal. Identification of candidate aptamer sequences conserved between orthologues across several plant species would certainly be an intriguing result.

### Reviewer 2: Rob Knight

In this manuscript, the authors propose that riboswitches might exist that bind cytokinins. The evidence in support of this contention is as follows. First, aptamers have been selected against a range of biological targets, including many molecules that are analogs of nucleotides or contain nucleotide bases as components. Second, riboswitches, which include an aptamer domain, are important agents for regulation in all three domains of life, although more examples have been found to date in the bacteria than in the other two domains. Third, cytokinins are adenine analogs, and are therefore plausible targets for RNA (or, indeed, DNA) aptamers to bind. Finally, expression analyses and binding site searches for known aptamers to an N6-substituted adenine shows that there are some transcripts that are regulated by cytokinins and also contain oligonucleotides matching the cores of these binding sites (although more extensive bioinformatics searches for an expression platform or, generally, alternate stable states, e.g. using the Vienna package or related tools, were not performed). The authors therefore propose that there are binding sites for cytokinins in Arabadopsis that are essentially the same as those identified by SELEX for related compounds. The main novelty claimed is that the hypothesis proposes that riboswtiches could be identified that bind small-molecules that are used as hormones, rather than closely related small-molecules that are used as metabolities, which could perhaps be seen as a somewhat arbitrary distinction.

Overall, the hypothesis is plausible, although I am not convinced that the hypothesis itself is sufficiently noteworthy to warrant publication as there are numerous classes of molecules that might plausibly be regulated by riboswitches but have not yet been shown to be so regulated in particular species or lineages. Additionally, substantially more statistical testing would be needed in order to rule out the possibility of chance matches in the binding site, and to show a specific association between transcripts containing the binding site and transcripts that are upregulated. I would strongly encourage the authors to experimentally test whether the transcripts they identify as containing the core motif actually bind adenine analogs (especially because the cost of synthesizing the relevant oligos with a T7 promoter is now so low) rather than publishing this as a hypothesis piece.

### Reviewer 3: Mikhail Gelfand

One of the most intriguing problem of riboswitches is the fact that none of them, to the exclusion of the THI-element (the TPP-binding riboswitch), have been observed outside bacteria. If not for the THI-element, one could easily assume that the riboswitch distribution is simply limited to the Eubacteria. Even THI-elements in the archeal Thermoplasma species could be explained by horizontal gene transfer from the Eubacteria. But the TPP riboswitches regulating splicing the plans and fungi seem ancient.

To address this problem, many groups repeatedly tried to identify new riboswitches in Eukaryotes, without obvious success. The authors of the hypothesis extend the search from metabolic enzymes to genes regulated by plant hormones, correctly noting that the chemical structure of the latter make it quite plausible that they could directly bind RNA aptamers.

The problem is, the bioinformatics analysis purported to corroborate the hypothesis seems insufficient. Firstly, the authors use as a search template SELEX-generated aptamers, while none of known natural riboswitches resembles in structure artificially synthesized aptamers for the same compounds. Secondly, the search pattern is rather weak and does not take into account secondary structure, hence leaving space for numerous false positives. Thirdly, no control analyses were made (e.g. with random patterns of the same information content and search conditions). Further, no structural rearrangements of the candidate riboswitch have been presented, nor a model for its interaction with the splicing-related motifs, nor analysis of its conservation in related species. Finally, the analysis of implications of changes in splicing and its consequences is based on rather weak evidence, again not supported by comparative genomic analysis.

Hence, while the hypothesis may be of interest, I feel that the presented analysis is insufficient as an independent corroboration, and essentially the papers remains a pure speculation.

## Author's Information

BD teaches a course in Advanced Molecular Biology. Preparations for this class gave rise to the insights discussed in this manuscript. JG was a student in this class and became interested in developing the idea further.
